# Supplementary material for: HELZ directly interacts with CCR4–NOT and causes decay of bound mRNAs
Source: Life Sci Alliance. 2019 Sep 30;2(5):e201900405. doi: 10.26508/lsa.201900405 (PMC6769256; doi:10.26508/lsa.201900405)

Hanet *et al.* Figure 1 source data

A Western blots Figure 1B and D

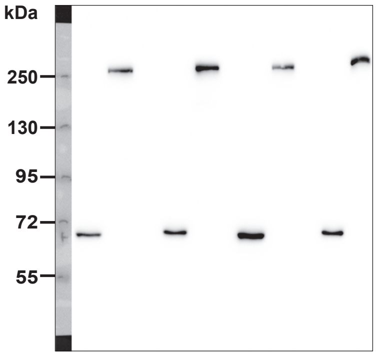

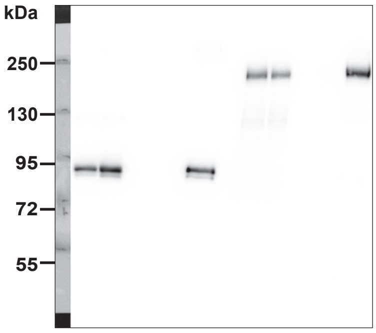

B Western Blots Figure 1C and E

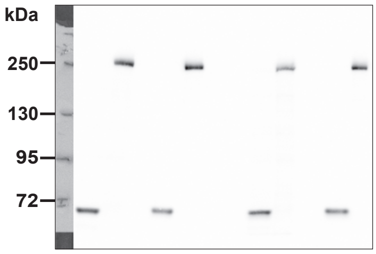

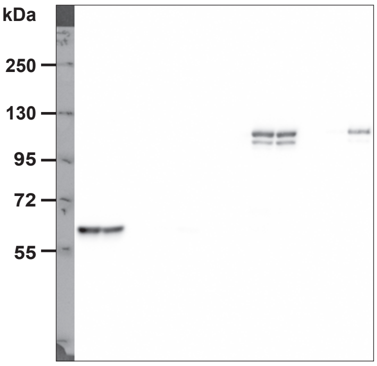

C Western Blots Figure 1F

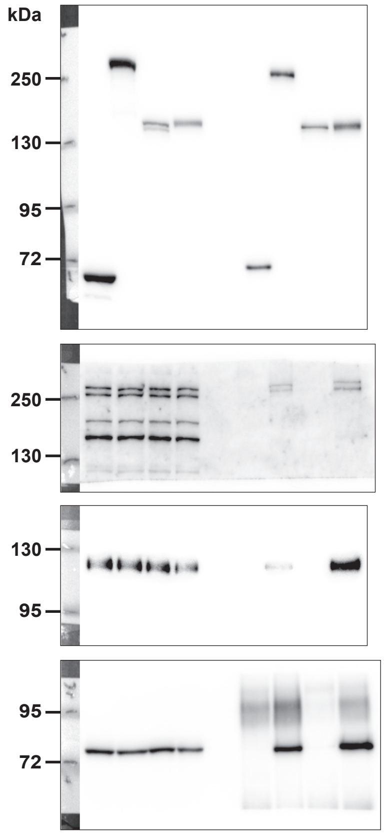

Hanet *et al.* Figure 2 source data

A Coomassie stained SDS-PAGE Figure 2B

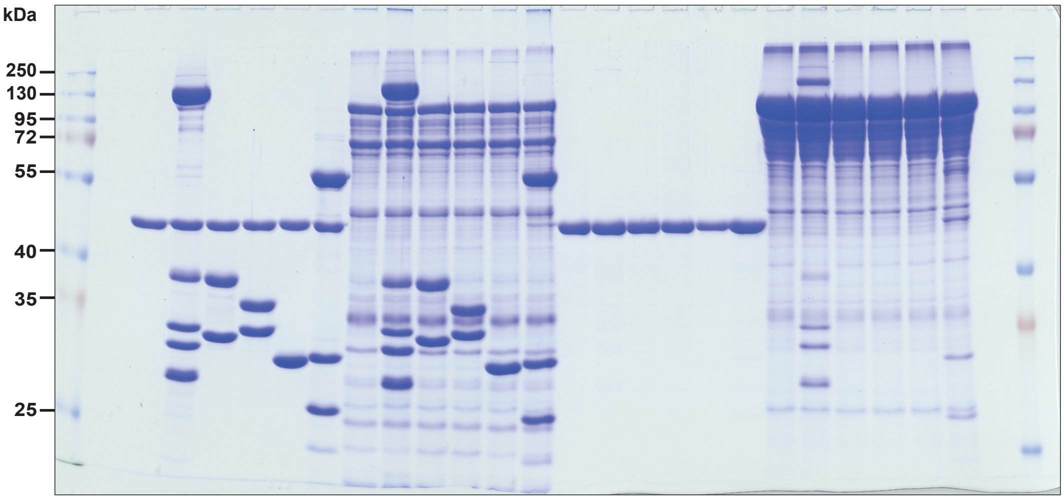

B Coomassie stained SDS-PAGE Figure 2C

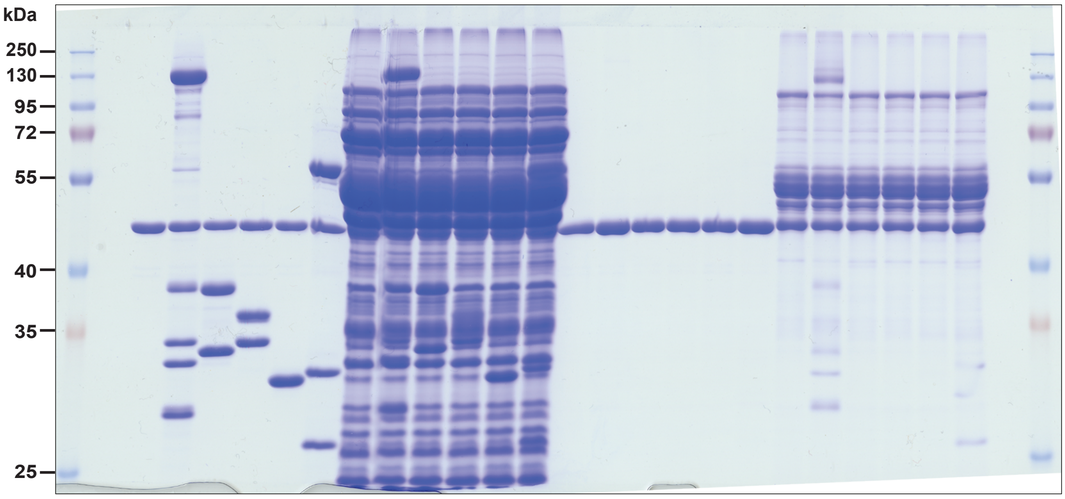

Hanet *et al.* Figure 3 source data

A NB Figure 3B

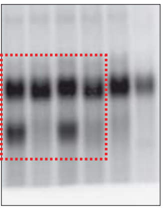

B WB Figure 3C

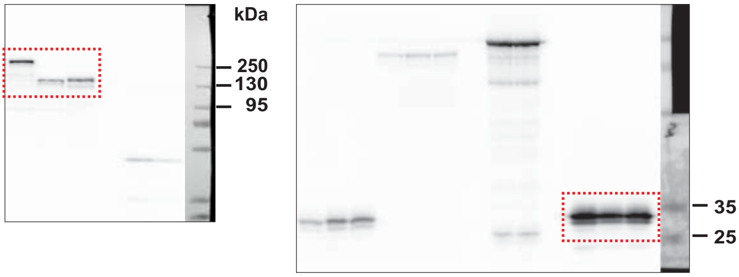

C WB Figure 3D

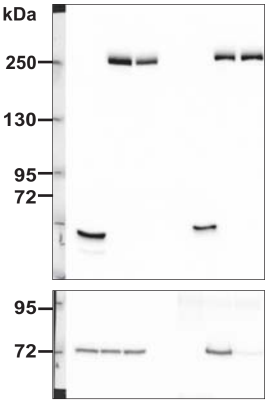

D NB Figure 3F

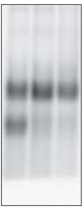

E WB Figure 3G

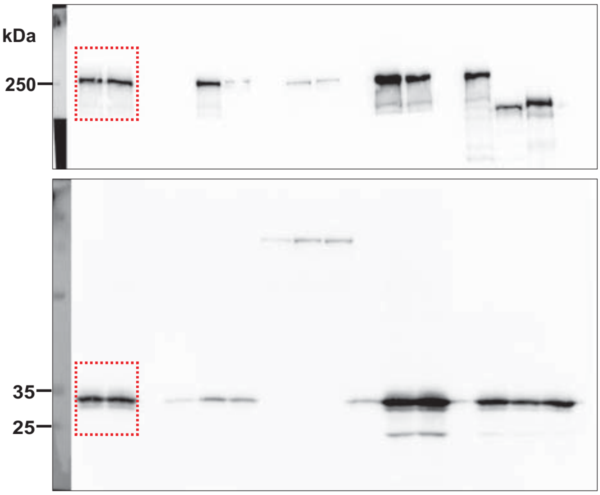

F NB Figure 3I

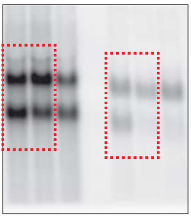

H NB Figure 3L

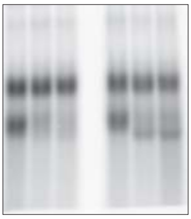

G WB Figure 3J

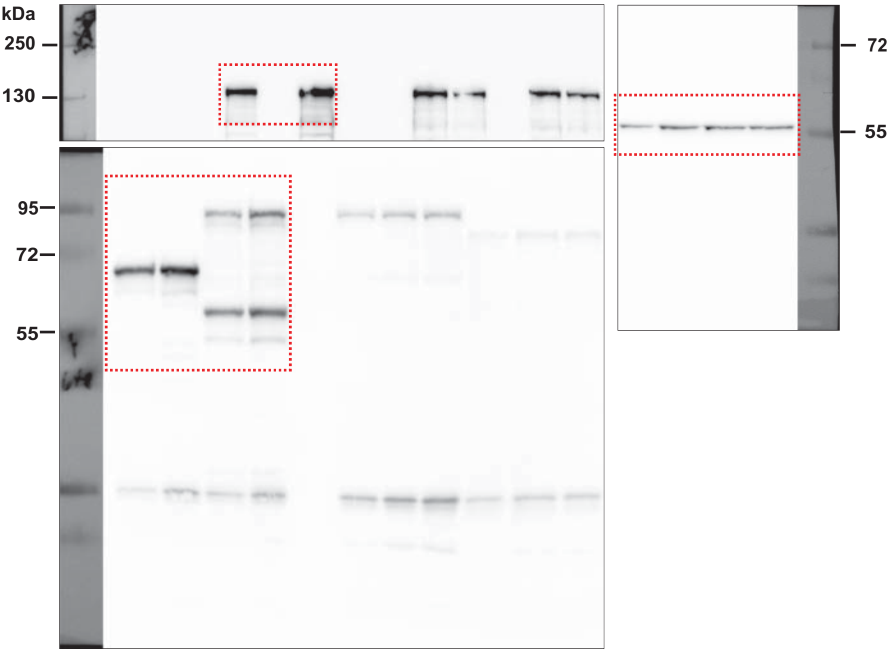

Hanet *et al.* Figure 3 source data

H WB Figure 3M

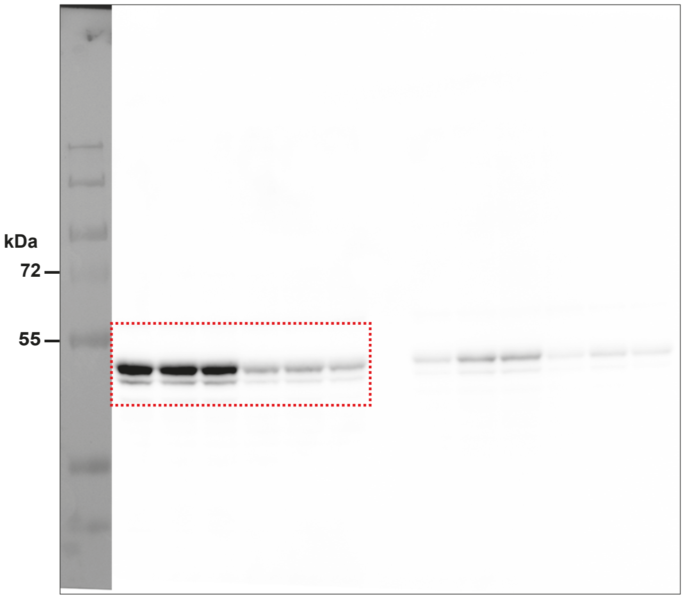

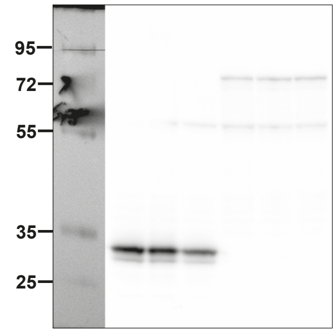

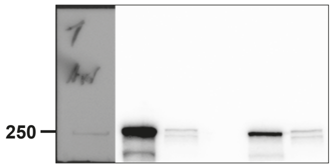

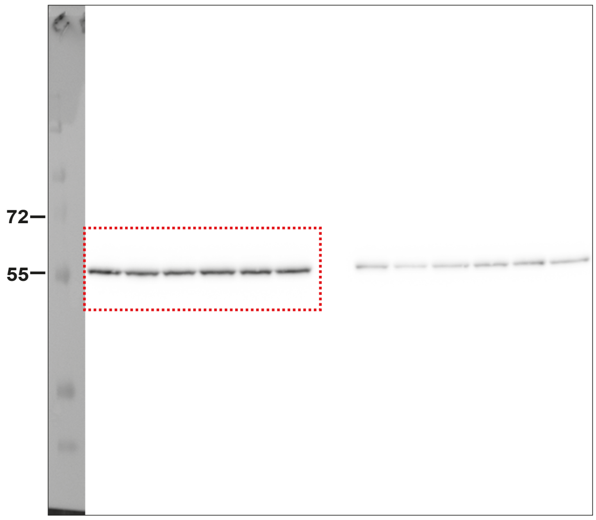

Hanet *et al.* Figure 4 source data

A WB Figure 4A to D

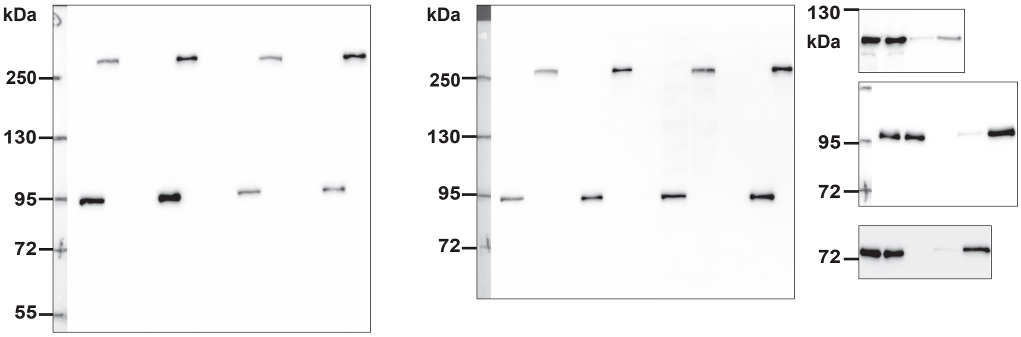

B NB Figure 4F

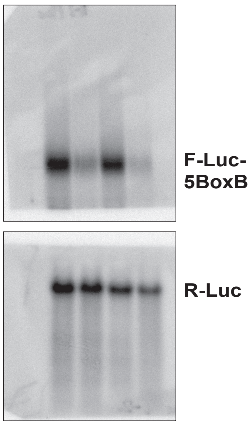

C WB Figure 4G

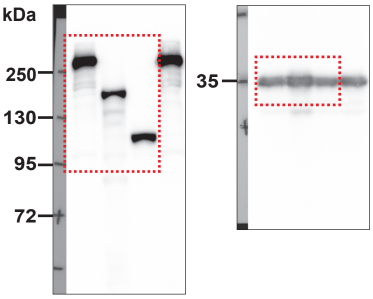

D WB Figure 4H

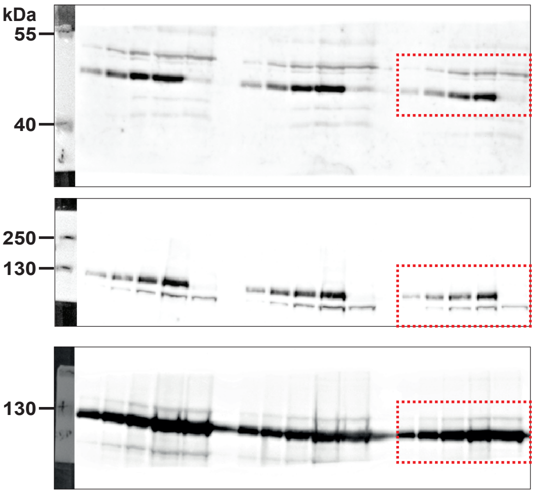

E NB Figure 4K

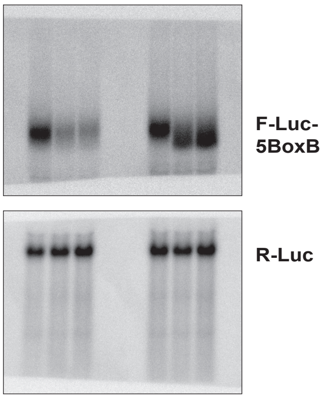

F WB Figure 4L

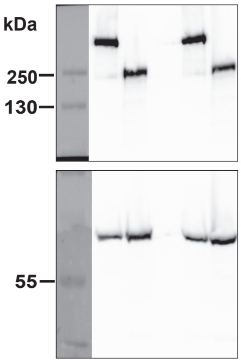

Hanet *et al.* Figure 5 source data

A NB Figure 5C

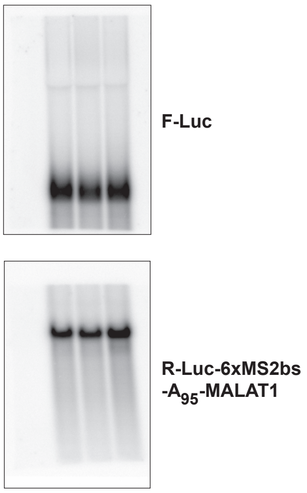

B NB Figure 5D

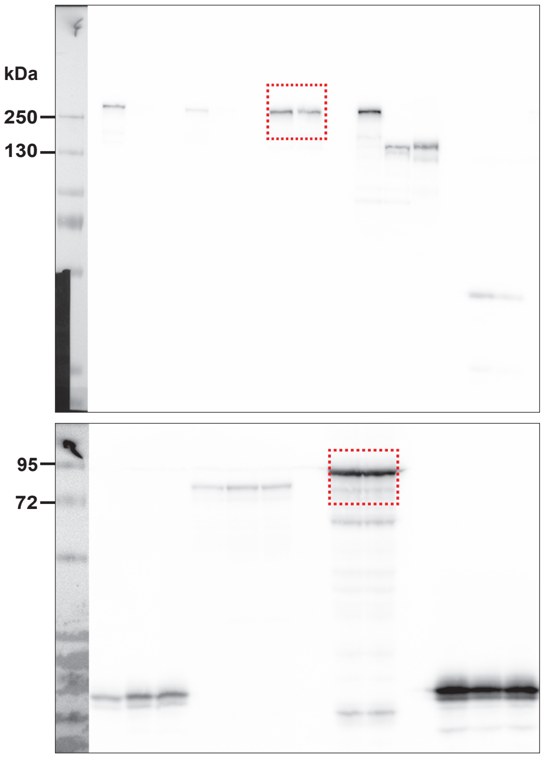

C WB Figure 5F

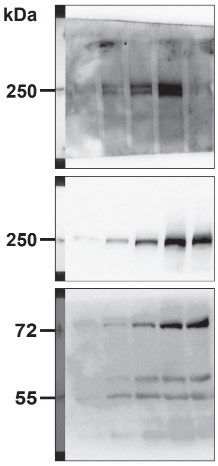

D WB Figure 5H

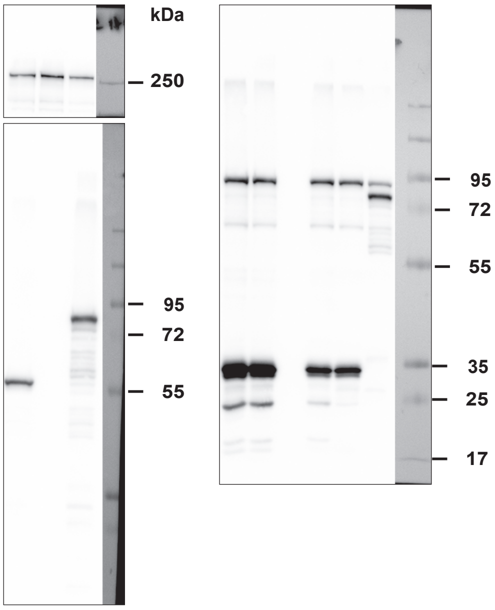

Supplement: Supplementary file 1 [file LSA-2019-00405_SdataF1-F5.pdf]
