# Supplementary material for: HELZ directly interacts with CCR4–NOT and causes decay of bound mRNAs
Source: Life Sci Alliance. 2019 Sep 30;2(5):e201900405. doi: 10.26508/lsa.201900405 (PMC6769256; doi:10.26508/lsa.201900405)
Supplement: Supplementary file 2 [file LSA-2019-00405_TableS1.doc]

**Table S1. Constructs used in this study.**

| ***Hs* HELZ (P42694)** | |
| --- | --- |
| Full-length | pT7-MS2-HA-HELZ M1-K1942 |
|  | pT7-GFP-HELZ M1-K1942 |
| HELZ-N | pT7-MS2-HA-HELZ M1-D1050 |
|  | pT7-GFP-HELZ M1-D1050 |
| HELZ-C | pT7-MS2-HA-HELZ P1051-K1942 |
|  | pT7-GFP-HELZ P1051-K1942 |
|  | pnEA-NvM-MBP-HELZ P1051-I1474-GB1-6xHis |
|  | pnEA-NvM-MBP-HELZ L1475-K1942-GB1-6xHis |
| F1107V (disrupts interaction with PABPC1) | pT7-MS2-HA-HELZ F1107V |
|  | pT7-GFP-HELZ F1107V |
| ***Dm* HELZ (CG9425)** | |
| Full-length | pAc5.1B-GFP-HELZ M1-Q2103 |
|  | pAc5.1B-λN-HA-HELZ M1-Q2103 |
| HELZ-N | pAc5.1B-λN-HA-HELZ M1-D1212 |
| HELZ-C | pAc5.1-λN-HA-HELZ P1213-Q2103 |
| ***Hs* mRNA decay factors** | |
|  | pλN-HA-C1-NOT1 |
| NOT1-Mid | pT7-GFP-C1-NOT1 M1085-T1605 |
| Catalytically inactive | pT7-GFP-C1-CAF1 D40A E42A |
| Catalytically inactive | pT7-GFP-C1-DCP2 E148Q |
|  | pCIneo-λN-HA-EDC4 |
|  | pλN-HA-C1-PatL1 |
|  | pλN-HA-C1-PAN3 |
|  | pCIneo-λN-HA-DDX6 |
|  | pT7-GFP-C1-DDX6 |
| ***Dm* mRNA decay factors** | |
|  | pAc5.1B-λN-HA-HPat |
|  | pAc5.1B-λN-HA-PAN3 |
|  | pAc5.1B-λN-HA-NOT1 |
|  | pAc5.1B-λN-HA-NOT2 |
|  | pAc5.1B-λN-HA-Ge-1 |
|  | pAc5.1B-λN-HA-Me31B |
