# Supplementary material for: HELZ directly interacts with CCR4–NOT and causes decay of bound mRNAs
Source: Life Sci Alliance. 2019 Sep 30;2(5):e201900405. doi: 10.26508/lsa.201900405 (PMC6769256; doi:10.26508/lsa.201900405)
Supplement: Supplementary file 5 [file LSA-2019-00405_TableS4.docx]

**Table S4.** Antibodies used in this study.

| **Antibody** | **Source** | **Catalog number** | **Dilution** | **Monoclonal/polyclonal** |
| --- | --- | --- | --- | --- |
| Anti-HA-HRP | Roche | 12013819001 | 1:5000 | Monoclonal |
| Anti-GFP (for WB) | Roche | 11814460001 | 1:3000 | Mouse monoclonal |
| Anti-GFP (for IP) | In house | - | - | Rabbit polyclonal |
| Anti-Tubulin | Sigma Aldrich | T6199 | 1:10 000 | Mouse Monoclonal |
| Anti-mouse IgG- HRP | GE Healthcare | NA931V | 1:10 000 | Sheep polyclonal |
| Anti-rabbit IgG-HRP | GE Healthcare | NA934V | 1:10 000 | Donkey polyclonal |
| Anti-*Hs* HELZ | Abnova | H00009931-M02 | 1:1000 | Mouse monoclonal |
| Anti-*Hs* PABPC1 | Abcam | Ab21060 | 1:5000 | Rabbit polyclonal |
| Anti-*Hs* NOT1 | In house | - | 1:1000 | Rabbit polyclonal |
| Anti-*Hs* NOT3 | Abcam | Ab55681 | 1:1000 | Mouse monoclonal |
| Anti-*Hs* DDX6 | Bethyl Laboratories | A300-461Z | 1:3000 | Rabbit polyclonal |
| Anti-*Hs* p70S6K (EDC4) | Santa Cruz Biotechnology | sc-8418 | 1:1000 | Mouse monoclonal |
| Anti-*Dm* DCP1 | In house | - | 1:1000 | Rabbit polyclonal |
| Anti-*Dm* Ge1 | In house | - | 1:1000 | Rat polyclonal |
| Anti-*Dm* PABP | In house | - | 1:1000 | Rabbit polyclonal |
