# Supplementary material for: HELZ directly interacts with CCR4–NOT and causes decay of bound mRNAs
Source: Life Sci Alliance. 2019 Sep 30;2(5):e201900405. doi: 10.26508/lsa.201900405 (PMC6769256; doi:10.26508/lsa.201900405)
Supplement: Supplementary file 6 [file LSA-2019-00405_TableS5.docx]

**Table S5. Oligos used in the qPCR reactions.**

| Name | Sequence |
| --- | --- |
| *sparc*_f | CTAGAGGCTCAGTGGTGGGA |
| *sparc*_r | TCCCTAGAGCCCCTGAGAAG |
| *basp1*_f | TGGATTTCCAAGATCCGCGT |
| *basp1*_r | TGGACAAGCTAAGTGGGCTC |
| *tenm1*_f | TCGCCTGATGGAACCCTCTA |
| *tenm1*_r | CCATTGCTGCTGGTAATCGC |
| *gapdh*_f | CTCTGCTCCTCCTGTTCGACAG |
| *gapdh*_r | TTCCCGTTCTCAGCCTTGACGG |
